# ABCC4 Variants Modify Susceptibility to Kawasaki Disease in a Southern Chinese Population

**DOI:** 10.1155/2018/8638096

**Published:** 2018-09-30

**Authors:** Di Che, Lei Pi, Zhenzhen Fang, Yufen Xu, Minmin Cai, Lanyan Fu, Huazhong Zhou, Li Zhang, Xiaoqiong Gu

**Affiliations:** ^1^Department of Clinical Biological Resource Bank, Guangzhou Institute of Pediatrics, Guangzhou Women and Children's Medical Center, Guangzhou Medical University, Guangzhou, China; ^2^Program of Molecular Medicine, Guangzhou Women and Children's Hospital, Zhongshan School of Medicine, Sun Yat-Sen University, Guangzhou, China; ^3^Department of Clinical Lab, Guangzhou Women and Children's Medical Center, Guangzhou Medical University, Guangzhou, China; ^4^Department of Cardiology, Guangzhou Women and Children's Medical Center, Guangzhou Medical University, Guangzhou, China

## Abstract

A previous family-based linkage study revealed that Kawasaki disease (KD) was associated with variations of the ATP-binding cassette subfamily C member 4 (ABCC4) gene in most European populations. However, significant differences exist among ethnic populations in European and Chinese subjects; therefore, whether ABCC4 variants indicate susceptibility to KD in Chinese children is unclear. The purpose of this research was to evaluate correlations between ABCC4 gene polymorphisms and susceptibility to KD in a Southern Chinese population. We genotyped six polymorphisms (rs7986087, rs868853, rs3765534, rs1751034, rs3742106, and rs9561778) in 775 KD patients and 774 healthy controls. Ninety-five percent confidence intervals (95% CIs) and odds ratios (ORs) were used to assess the strength of each association. We found that the rs7986087 T variant genotype was associated with significantly higher susceptibility to KD (adjusted OR = 1.30, 95% CI = 1.05–1.60 for rs7986087 CT/TT). However, the rs868853 T variant genotype was associated with significantly lower susceptibility to KD (adjusted OR = 0.74, 95% CI = 0.59–0.92 for rs868853 CT/CC). Compared with the patients with 0–4 ABCC4 risk genotypes, the patients with 5-6 ABCC4 risk genotypes had a significantly increased risk of KD (adjusted OR = 1.63, 95% CI = 1.07–2.47), and this risk was more significant in the subgroups of females, subjects aged 12–60 months, and individuals with coronary artery lesions. These results indicate that specific single-nucleotide polymorphisms in the ABCC4 gene may increase susceptibility to KD in a Southern Chinese population.

## 1. Introduction

Kawasaki disease (KD) manifests as acute systemic vasculitis in medium-sized arteries and primarily affects infants and children under five years of age [1]. It is the leading cause of childhood-acquired heart disease in the developed world, and up to 25% of affected patients, if not adequately treated with intravenous immunoglobulin, will develop coronary artery lesions (CALs), including coronary artery dilation or aneurysm formation [2, 3]. The incidence rate of KD was previously reported to be as high as 239.6 per 100,000 children under five years old in Japan but only approximately 49.4 per l00,000 children under five years old in China [4]. The incidence rate of patients with KD has been continuously increasing all over the region, including in Japan, Korea, and China [5–7]. The cause of the disease remains unknown. However, as a result of several decades of extensive international investigation, a wealth of reports has indicated that genetic and environmental factors might play a critical role in the development of KD in susceptible hosts.

A genome-wide association study (GWAS) is the primary method used to identify hereditary genetic variation related to multiple complex human diseases, such as KD [8–11]. Previous GWASs have confirmed that several loci confer susceptibility to KD, including single-nucleotide polymorphisms (SNPs) in the BLK [12, 13], FCGR2A [14, 15], ITPKC [14, 16], and CASP3 [17, 18]. Khor et al. found that ABCC4 gene polymorphisms were associated with susceptibility to KD in European populations in a family-based linkage study performed using a cohort of 1284 KD subjects and their family members (a total of 3248 individuals) [19]. Moreover, oral aspirin was the first-line treatment for KD, and the antiplatelet effects of aspirin for healing endothelial dysfunction and preventing clot formation were necessary to decrease the occurrence of coronary artery complications [20, 21]. ABCC4 is a potential pharmacologic target for cardiovascular disease, via its vasodilator and antiplatelet effects [22]. These data suggest that ABCC4 may affect immune activation and the vascular response to injury observed in KD pathogenesis. However, significant differences exist among ethnic populations that include European and Chinese subjects, and these differences contribute to the statistical significance of genetic associations and may therefore alter the interrelation between SNPs and diseases, including KD [23]. Therefore, in this case-control study (775 cases and 774 controls), two SNPs in the ABCC4 gene (rs7986087 and rs868853) that were found to be associated with KD susceptibility in most European populations and another four SNPs (rs3765534, rs1751034, rs3742106, and rs9561778) that were found to be associated with immune-related diseases and drug resistance were selected [24–27]. We attempted to clarify the existence and nature of the relationship between ABCC4 gene polymorphisms and susceptibility to KD in a Chinese population. Our study may promote further investigation of the pathogenesis of KD in Chinese subjects.

## 2. Materials and Methods

### 2.1. Study Subjects

A total of 775 patients with KD were enrolled in the study. They were diagnosed according to the criteria of the American Heart Association [1]. All patients were unrelated ethnic Chinese Han individuals who were recruited from the Guangzhou Women and Children's Medical Center, mainly between February 2010 and March 2016. Additionally, 774 age- and gender-matched healthy controls were randomly selected from children undergoing a physical examination during the same period. Written informed consent was obtained from every participant or his/her guardian. This study was performed with authorization from the Institutional Committee of Guangzhou Women and Children's Medical Center (2015090113). Each participant donated 2 mL of blood for genomic DNA extraction.

### 2.2. SNP Selection and Genotyping

We selected SNPs of interest using the NCBI SNP (https://www.ncbi.nlm.nih.gov/snp/) and kb SNP (http://www.ncbi.nlm.nih.gov/) databases using the following three standards: (1) the SNP was located in the regulatory region of a gene (i.e., in an exon, the 5′ region near the gene, the 5′ untranslated region [UTR], the 3′ UTR, or the 3′ region near the gene and splice sites); (2) the minor allele frequency (MAF) was reported in HapMap in Southern Chinese descendants and was not less than 5%; (3) the SNP affected microRNA binding site activity or transcription factor binding site activity at the putative promoter region or changed the exon amino acid sequence. The selected SNPs satisfied most of these criteria. These included two SNPs (rs7986087 and rs868853) in the ABCC4 gene that were identified in a previous genome-wide linkage study. We performed multiplex PCR to genotype these SNPs. Then, high-quality genomic DNA samples were genotyped by PCR using multiple gene-specific primer pairs. This step enriched the specific SNPs and, when used together with indexing primers, allowed massive parallel sequencing using an Ion Proton system (Life Technologies).

### 2.3. Statistical Analysis

The genotype frequencies at each locus and the demographic variables (e.g., gender and age) were compared between the healthy controls and the KD patients by using the *χ*^2^ test. In the controls, the genotypic frequency of each SNP was tested for departure from the Hardy-Weinberg equilibrium (HWE) using the goodness-of-fit *χ*^2^ test. The associations between the selected SNPs and susceptibility to KD were determined by calculating 95% confidence intervals (CIs) and odds ratios (ORs) using unconditional logistic regression analyses. Stratified analyses were performed by subgroups, including age, gender, and coronary artery aneurysms (CAAs) or coronary artery lesions (CALs). The patients were stratified by age after adjusting for gender or stratified by gender after adjusting for age. According to the Japanese Kawasaki Disease Research Committee, CAA and CAL were defined by absolute diameters or *z*-scores as previously described [1, 28]. All statistical analyses were performed in SAS software (Version 9.1; SAS Institute, Cary, NC, USA). *P* values less than 0.05 were considered statistically significant [29].

## 3. Results

### 3.1. Characteristics of the Population

A total of 775 KD patients and 774 gender-, age-, and ethnicity-matched controls were included in the present study (Table 1). No significant differences were observed in gender (*P* = 0.9274) and age (29.11 ± 24.86 vs. 25.65 ± 23.30 months old, *P* = 0.2228) between the KD patients and healthy controls. Moreover, among the KD patients, 103 (13.29%) developed CAA, 187 (24.13%) developed CAL, and 485 (62.58%) developed non-coronary artery lesions (NCALs).

### 3.2. Associations between ABCC4 Gene Polymorphisms and Susceptibility to Kawasaki Disease

All observed genotype frequency distributions among the six SNPs were in accordance with HWE in the control subjects. Among the six investigated SNPs, significant differences were found in the genotype distributions for rs7986087 T (*P* = 0.0197) and rs868853 T (*P* = 0.0256) between the KD patients and the healthy controls (Table 2).

After adjusting for age and gender, when the rs7986087 CC genotype was used as the reference, the T variant genotypes were associated with an increased risk of KD (adjusted OR = 1.37, 95% CI = 1.09–1.70 for CT; adjusted OR = 0.90, 95% CI = 0.55–1.48 for TT; and adjusted OR = 1.30, 95% CI = 1.05–1.60 for CT/TT). When the rs868853 CC genotype was used as the reference, the T variant genotypes were associated with a decreased risk of KD (adjusted OR = 1.28, 95% CI = 0.56–2.54 for CT; adjusted OR = 0.92, 95% CI = 0.48–1.80 for TT; and adjusted OR = 0.74, 95% CI = 0.59–0.92 for CT/CC). However, the risk of KD was not associated with the other four SNPs. A combination analysis showed that the risk of KD was significantly higher in individuals with 5-6 risk genotypes than in individuals with 0–4 risk genotypes (adjusted OR = 1.63, 95% CI = 1.07–2.47, *P* = 0.0229).

### 3.3. Stratification Analysis

We further determined the associations between variant genotypes of six selected SNPs in the ABCC4 gene and the risk of KD by stratifying the subjects by subgroups, including age, sex, and coronary artery outcomes (Table 3). When all risk genotypes were combined into a new variable, we found that compared with individuals carrying 0–4 risk genotypes, those carrying 5-6 risk genotypes had a higher risk in the following groups: individuals aged 12 to 60 months old (adjusted OR = 1.65, 95% CI = 1.05–2.59), females (adjusted OR = 2.69, 95% CI = 1.43–5.03), and individuals with NCALs (adjusted OR = 1.77, 95% CI = 1.18–2.63). However, there was no difference in risk when patients were stratified based on the presence of coronary artery aneurysm (CAA) formation.

## 4. Discussion

In the present case-control study, which included 775 KD patients and 774 healthy controls, we show that the ABCC4 gene rs7986087 T variant genotype was correlated with increased susceptibility to KD. However, the rs868853 T variant genotype was associated with decreased susceptibility to KD. To the best of our knowledge, this is the first study to investigate the association between ABCC4 gene polymorphisms and susceptibility to KD in Southern Chinese children.

ABCC4, also known as MRP4, is a large gene (282 kb) [30, 31]. The ABCC4 protein is expressed in a variety of organs, including the brain [32], lungs [33], kidneys [34], prostate [35], and liver [30], and in platelets [36]. It has been shown to play a significant role in the release of prostaglandins from cells. In particular, ABCC4 is unique because it mediates the efflux of PGE1 and PGE2 to inhibit the anti-inflammatory effect of nonsteroidal agents in vitro [30]. Additionally, studies have indicated that the ABCC4 gene is involved in the ATP-dependent efflux of acyclic nucleoside monophosphates, such as the anti-HIV drugs 9-(2-phosphonylmethoxyethyl) adenine (PMEA) and 9-(2-phosphonylmethoxyethyl) guanine [37–39]. Many studies have found that variants of ABCC4 were associated with multiple disease susceptibilities. Palikhe et al. found that the variants of ABCC4 (rs868853) were associated with airway inflammation in asthmatics [40], and Likanonsakul et al. found that the ABCC4 4976C allele was associated with beta2-microglobulinuria in human immunodeficiency virus- (HIV-) infected patients in Thailand and showed that this difference may help identify patients at greater risk of developing tenofovir diisopropyl fumarate-induced kidney tubular dysfunction [41]. Variants of ABCC4 (rs9561778) showed a significant association with cyclophosphamide-induced adverse drug reactions in breast cancer patients [27]. Low et al. confirmed that ABCC4 rs9561778 gene polymorphisms might predict the risk of adverse drug reactions (ADRs) in patients receiving cyclophosphamide (CPA) combination chemotherapy [27].

GWAS is an efficient method to identify inherited genetic variations that are associated with complex human diseases [8, 42]. In a GWAS that included 250 KD patients and 446 healthy controls in a Han Chinese population residing in Taiwan, three SNPs in the gene for the coatomer protein complex beta-2 subunit and six SNPs in the gene for endoplasmic reticulum aminopeptidase 1 were found to be associated with an increased risk of KD [43]. Another GWAS and replication analysis of five independent sample populations collected from around the world that included 2173 individuals with KD and 9383 controls found that the FCGR2A gene was associated with higher susceptibility to KD [14]. A genetic analysis of ABCC4 found that the ABCC4 protein may be responsible for prostaglandin transport and augmenting inflammation during KD pathogenesis [19]. Resequencing studies of ABCC4 are warranted to increase our understanding of the risk alleles associated with susceptibility to KD. ABCC4 genetic variants are very common, and the presence of two risk alleles further increased the risk of KD [19]. Therefore, improving our knowledge of this gene's function in KD may provide important insight into pathogenesis. Khor et al. [19] genotyped 35 SNPs in a total of 1284 KD patients and their family members (a total of 3248 individuals). In all, 10 of the 35 SNPs were associated with susceptibility to KD.

Khor et al. observed that the rs7986087 T variant genotype significantly increased KD susceptibility in a European population. Notably, in our study, we observed similar results in Southern Chinese children. In contrast, we found that rs868853 T variant genotypes decreased KD susceptibility in Southern Chinese children, unlike the research of Khor et al., in which no associations with KD susceptibility were found. These findings suggest that rs868853 T variant genotypes may be more race-specific at the genetic level. However, it is not clear why ABCC4 rs868853 variants play opposite effects on susceptibility to KD. This is also a limitation of our research.

When the six ABCC4 SNPs were analyzed, we found that patients with 5 or more KD risk genotypes were significantly more likely to develop the disease than were those with only 0–4 of the risk genotypes. The population with the highest incidence of KD was previously reported to be children between 6 months and 5 years of age in many countries around the world [44]. Additionally, in Italy, the male/female ratio among KD patients was 1.4 : 1 [45]. However, in the stratified analysis, we found that in two groups, including individuals aged 12 to 60 months old and females, those carrying 5-6 risk-associated genotypes had a higher risk of developing the disease. We hypothesized that ABCC4 SNPs may be associated with the male-to-female ratio in KD. Females carrying 5-6 risk-associated genotypes had significantly more susceptibility to KD than males, suggesting that ABCC4 gene polymorphisms may modify the risk of males suffering from KD. KD preferentially involves the coronary arteries and can lead to dilatation, aneurysm, subsequent coronary artery thrombosis, or myocardial infarction [3]. However, there was no difference in the risk of developing the disease when patients were stratified by CAA formation.

Although this is the first study to investigate ABCC4 genetic polymorphisms in Southern Chinese children with Kawasaki disease, the limitations of this study should be understood. First, the current sample size was not large enough. Multicenter studies with larger sample sizes are required to validate the relationship between ABCC4 gene polymorphisms and susceptibility to KD. Second, we evaluated only six ABCC4 SNPs that were identified in a previous genome-wide linkage study. No other potentially functional SNPs were investigated. Additional potentially functional ABCC4 gene polymorphisms should be investigated. Finally, this study was retrospective; only gender and age were adjusted in the logistic regression analysis. In addition, we were unable to collect or control for other factors, such as dietary intake or environment exposure in the parents and children.

In summary, the results of the present study confirm that in a Southern Chinese population, the ABCC4 gene polymorphism rs7986087 T variant genotype is associated with significantly higher susceptibility to KD, whereas the rs868853 T variant genotype was associated with significantly lower susceptibility to KD. Increasing our knowledge of this gene's function in KD may provide valuable insights into its role in the disease pathogenesis. However, multicenter studies that involve practical experiments and have larger sample sizes should be performed to further characterize ABCC4 and determine the mechanisms underlying its role in KD.

## Figures and Tables

**Table 1:** Frequency distribution of selected characteristics in Kawasaki disease patients and healthy controls.

Variables	Patients (*n* = 775)		Controls (*n* = 774)		*P* ^a^
	No.	%	No.	%	
Age range, month	1–166		1–144		0.2228
Mean ± SD	29.11 ± 24.86		25.65 ± 23.30		
<12	185	23.87	177	22.87	
12–60	527	68	550	71.06	
>60	63	8.13	47	6.07	
Gender					
Male	524	67.61	525	67.83	0.9274
Female	251	32.39	249	32.17	
*Coronary artery outcomes*					
CAA	103	13.29			
CAL	187	24.13			
NCAL	485	62.58			

^a^Two-sided *χ*^2^ test for distributions between Kawasaki disease patients and controls.

**Table 2:** Genotype frequency distribution of ABCC4 in KD patients and healthy controls.

Genotype	Patients (*n* = 775)	Controls (*n* = 774)	*P* value^a^	OR (95% CI)	*P* value	Adjusted OR (95% CI)	*P* value^b^
*ABCC4/rs7986087 C > T (HWE = 0.5938)*							
CC	488 (62.97)	530 (68.48)	**0.0197**	1.000		1.000	
CT	257 (33.16)	207 (26.74)		**1.35 (1.08–1.68)**	**0.0079**	**1.37 (1.09–1.70)**	**0.0057**
TT	30 (3.87)	37 (4.78)		0.88 (0.54–1.45)	0.6160	0.90 (0.55–0.48)	0.6794
Dominant	287 (37.03)	244 (31.52)	**0.0223**	**1.28 (1.03–1.58)**	**0.0225**	**1.30 (1.05–1.60)**	**0.0161**
Recessive	745 (96.13)	737 (95.22)	0.0786	0.80 (0.49–1.31)	0.3815	0.82 (0.50–1.34)	0.4193
*ABCC4/rs3742106 A > C (HWE = 0.8429)*							
AA	171 (22.06)	191 (24.68)	0.1618	1.000		1.000	
AC	389 (50.19)	399 (51.55)		1.09 (0.85–1.40)	0.5026	1.09 (0.85–1.40)	0.4829
CC	215 (27.74)	184 (23.77)		1.30 (0.98–1.74)	0.0672	1.32 (0.99–1.76)	0.0547
Dominant	604 (77.93)	583 (75.32)	0.2243	1.16 (0.91–1.46)	0.2250	1.17 (0.92–1.48)	0.2032
Recessive	560 (72.25)	590 (76.23)	0.0740	1.23 (0.98–1.55)	0.0747	1.25 (0.99–1.57)	0.0608
*ABCC4/rs9561778 G > T (HWE = 0.3799)*							
GG	381 (49.16)	410 (52.97)	0.2779	1.000		1.000	
GT	332 (42.84)	312 (40.31)		1.14 (0.93–1.41)	0.2023	1.14 (0.93–1.41)	0.2147
TT	62 (8)	52 (6.72)		1.28 (0.86–1.90)	0.2160	1.25 (0.84–1.86)	0.2625
Dominant	394 (50.84)	364 (47.03)	0.1336	1.16 (0.95–1.42)	0.1339	1.16 (0.95–1.41)	0.1519
Recessive	713 (92)	722 (93.28)	0.3338	1.21 (0.82–1.77)	0.3358	1.18 (0.80–1.73)	0.3950
*ABCC4/rs1751034 C > T (HWE = 0.6842)*							
CC	36 (4.65)	34 (4.39)	0.8256	1.000		1.000	
CT	271 (34.97)	282 (36.43)		0.91 (0.55–1.49)	0.7026	0.94 (0.57–1.56)	0.8223
TT	468 (60.39)	458 (59.17)		0.96 (0.59–1.57)	0.8860	1.01 (0.61–1.63)	0.9932
Dominant	739 (95.36)	740 (95.6)	0.8110	0.94 (0.58–1.52)	0.8111	0.98 (0.60–1.59)	0.9346
Recessive	307 (39.62)	316 (40.82)	0.6262	1.05 (0.86–1.29)	0.6261	1.05 (0.86–1.29)	0.6104
*ABCC4/rs3765534 C > T (HWE = 0.5252)*							
CC	701 (90.45)	677 (87.47)	0.1689	1.000		1.000	
CT	73 (9.42)	96 (12.4)		0.73 (0.53–1.01)	0.0604	0.74 (0.53–1.02)	0.0666
TT	1 (.13)	1 (.13)		0.97 (0.06–15.47)	0.9804	0.73 (0.04–12.14)	0.8294
Dominant	74 (9.55)	97 (12.53)	0.0606	0.74 (0.53–1.01)	0.0617	0.74 (0.54–1.02)	0.0650
Recessive	774 (99.87)	773 (99.87)	0.9993	1.00 (0.06–16.02)	1.0000	0.75 (0.04–12.43)	0.8421
*ABCC4/rs868853 C > T (HWE = 0.2545)*							
CC	18 (2.32)	18 (2.33)	**0.0256**	1.000		1.000	
CT	228 (29.42)	181 (23.39)		1.26 (0.64–2.49)	0.5070	1.28 (0.65–2.54)	0.4749
TT	529 (68.26)	575 (74.29)		0.92 (0.47–1.79)	0.8056	0.92 (0.48–1.80)	0.8190
Dominant	757 (97.68)	756 (97.68)	0.9969	1.00 (0.52–1.94)	0.9969	1.01 (0.52–1.96)	0.9764
Recessive	246 (31.74)	199 (25.72)	**0.0087**	**0.74 (0.59–0.93)**	**0.0088**	**0.74 (0.59–0.92)**	**0.0067**
*Combined effect of risk genotypes*							
0–4	691 (89.16)	722 (93.28)		1.000		1.000	
5-6	84 (10.84)	52 (6.72)	0.0040	**1.63 (1.07–2.47)**	**0.0217**	**1.63 (1.07–2.47)**	**0.0229**

^a^ *χ* ^2^ test for genotype distributions between Kawasaki disease patients and controls; ^b^adjusted for age and gender.

**Table 3:** Stratification analysis of susceptibility in Kawasaki disease patients.

Variables	Combined effect of risk genotypes (patients/controls)		*P* value^a^	OR (95% CI)	*P* value	Adjusted OR (95% CI)	*P* value^b^
	0–4	5-6					
*Age, months*							
<12	163/162	22/15	0.2818	1.46 (0.73–2.91)	0.2854	1.46 (0.73–2.91)	0.2855
12–60	475/516	52/34	0.0253	**1.66 (1.06–2.61)**	**0.0270**	**1.65 (1.05–2.59)**	**0.0296**
>60	53/44	10/3	0.1155	2.77 (0.72–10.68)	0.1397	3.52 (0.87–14.33)	0.0790
*Gender*							
Male	477/488	47/37	0.2510	1.30 (0.83–2.04)	0.2526	1.31 (0.84–2.06)	0.2378
Female	214/234	37/15	0.0012	**2.70 (1.44–5.05)**	**0.0020**	**2.69 (1.43–5.03)**	**0.0021**
*Coronary artery outcomes*							
CAA	91/722	12/52	0.0904	1.83 (0.94–3.56)	0.0744	1.84 (0.94–3.60)	0.0748
CAL	170/722	17/52	0.2727	1.39 (0.78–2.46)	0.2609	1.39 (0.78–2.47)	0.2619
NCAL	430/722	55/52	0.0047	**1.78 (1.19–2.64)**	**0.0046**	**1.77 (1.18–2.63)**	**0.0053**

^a^ *χ* ^2^ test for genotype distributions between Kawasaki disease patients and controls; ^b^adjusted for age and gender.

## Data Availability

The data used to support the findings of this study are included within the article.

## Abbreviations

NCAL: Non-coronary artery lesions
CAL: Coronary artery lesions
CAA: Coronary artery aneurysm
ABCC4: ATP-binding cassette subfamily C member 4
GWAS: Genome-wide association study.
